# Supplementary material for: Indirect interactions influence contact network structure and diffusion dynamics
Source: R Soc Open Sci. 2019 Aug 28;6(8):190845. doi: 10.1098/rsos.190845 (PMC6731728; doi:10.1098/rsos.190845)
Supplement: Indirect interactions influence contact network structure and diffusion dynamics - Supplementary material [file rsos190845supp1.pdf]

Indirect interactions influence contact network structure and diffusion dynamics

SUPPLEMENTARY MATERIAL

Md Shahzamal[1,2], Raja Jurdak[2,1], Bernard Mans[1] and Frank de Hoog[2]

[1]Department of Computing, Macquarie University, Sydney, Australia
[2]Data61, Commonwealth Scientific and Industrial Research Organization (CSIRO), Australia

This electronic supplementary material provides more details of the applied methods and results that support the arguments presented in the main text. The applied empirical individual contact networks are built based on the location updates made by the users of social networking application called Momo. The processing and extraction procedures of possible direct and indirect transmission links are discussed in the section **S1**. We have presented the concise version of SPDT infection risk assessment model in the main test. We elaborate the mathematical development of our developed infection risk assessment model in supplementary material section **S2**. Finally, the details simulation results are presented in the section **S3** to clarify some arguments of main text.

Contents

S1 Empirical contact networks **2**
- S1.1 Transmission link extraction 2
- S1.2 Networks properties 2
- S1.3 Various network configurations 4

S2 SPDT risk assessment model **5**
- S2.1 Mathematical formulation 5
- S2.2 Characterizing model 7

S3 Simulation and Analysis **10**
- S3.1 Impacts of particle removal rates 10
- S3.2 Impacts of biological disease parameters 15

S1 Empirical contact networks

S1.1 Transmission link extraction

All possible disease transmission links according to SPDT diffusion model definition are extracted from location updates of Momo users. Around 56 million location updates of 364K users from Beijing city are applied in our work. These location updates are made over 32 days. To create an SPDT link between a host user v (assume infected with disease) and a neighbor user u (assume susceptible), it is required to find the arrival times (t_s, t'_s) and departure times (t_s, t'_s) of host and neighbour users respectively at the interaction location.

As the first step, it is identified that an infected host user v is staying at a location. Consecutive updates, $X = \{(x_1, t_1), (x_2, t_2), \ldots (x_k, t_k)\}$ where x_i are the co-ordinate values and t_i are the update times, from a user v within a radius of 20m (travel distance of airborne infection particles [1]) of the initial update's location x_1 are indicative of the user staying within the same proximity of x_1. A threshold is set for time difference of any two updates to 30 minutes to make sure infected host remain within the same proximity, as longer gaps may indicate a data gap in the user movement's pattern. Then, the central co-ordinate in the update X is searched where the distances from each update to all other updates are added together and the update x_c with the minimum sum is taken as the central co-ordinate. For the host user v, its visit to the proximity of x_c will represent a valid visit if a susceptible user u has location updates starting at $t_1^{'}$ while v is present, or within δ seconds (assume $\delta = 200$ min while sufficient particles will remain at the location) after v leaves the interaction location. The neighbour susceptible user u should have at least two updates within 20m of x_c to be valid and ensure that it is in fact staying at the same proximity, and therefore can be exposed to the infected particles, rather than simply passing by. The stay period of host user v at the proximity of x_c is $(t_s = t_1, t_l = t_k)$, where t_k represents the end of the current stay period. If u's last update within 20m around x_c is $(x_j^{'}, t_j^{'})$, the created SPDT link has a link duration $(t'_s = t'_1, t'_l = t_j^{'})$ due to active visit $(t_s = t_1, t_l = t_k)$ of v. All links to other users for this active visit $(t_s = t_1, t_l = t_k)$ are computed. In the similar way, all visits made by v are searched over the updates of 32 days and SPDT links with all neighbours are extracted. This process are executed for all users present in the data set to find the all possible SPDT disease transmission links and provides a real contact network with SPDT links among users. A SPDT link is noted as $e_{vu} = (v(t_s, t_l), t'_s, t'_l)$ which means that a user v visits a location during (t_s, t_l) where another user u is present for the duration (t'_s, t'_l) and obviously $t'_s \geq t_s$. Each link between two specific users are distinguished by the time intervals (t_s, t_l) and (t'_s, t'_l). Finally, we have obtained a SPDT network of 364K users [2].

S1.2 Networks properties

In our main text, we have described the degree distribution and clustering co-efficient properties of SPDT network. Here, we also analysis some other features of the SPDT

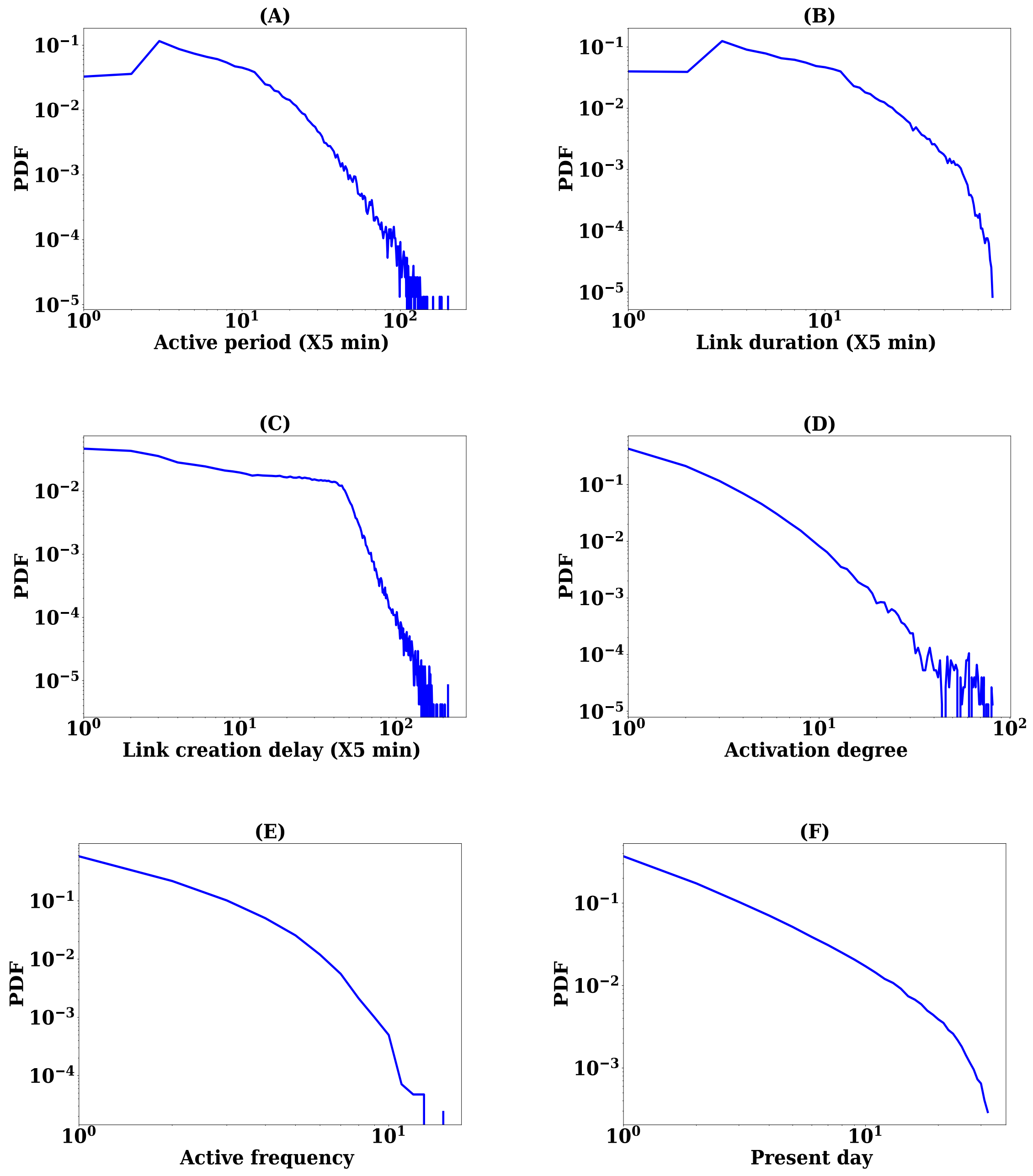

Figure S1: Properties of the extracted SPDT network: A) active period, B) link duration, C) link creation delay, D) activation degree, E) activation frequency and F) users present day

contact network. For each visit to a location, a user stays for a period of time called active period $(t_l - t_s)$. The Fig. S1A shows the distribution of active period lengths collected over the seven days of the constructed network. The distribution of active periods have the similar trends of exponential distribution. The authors [3, 4] have shown that the periods that individuals stay at the locations follows a distribution with long tail. The average time users staying at a location is about 56 min. During this period infected individual can deposit the significant infectious particles at the visited locations. We also look at the link duration (the time susceptible individuals are staying at the host's

Table S1: Values for various networks

Networks	SDT	SST	DDT	DST	LDT	LST
Total links (M)	6.86	2.10	47.11	13.24	47.11	47.11
Connected users (K)	364	264	364	264	364	364
Link density	18.86	7.95	129.42	50.15	129.42	129.42

location). The link duration is shown in the Fig. S1B and it is following the same behaviours of the active period. The average link duration is about 50 min. This period would be sufficient to inhale infectious particles capable of causing infectious disease. We have seen in the above link processing that susceptible individual may arrive after the infected individual's arrival. This has a strong impact on the spreading process. Thus, we also study the link creation delay after the infected individual arrive. The distribution of link creation delays is shown in the Fig. S1C. The average link creation delay is about 100 min which is the time difference between the arrival of host and neighbor individuals at the interaction location. Another interesting property is that the number of users a given user interacts with when it is active. We define this as the activation degree. The activation degree is presented in the Fig. S1D. This is following the power-law distribution which is found in many real world scenarios [5, 6]. The SPDT network assumes the properties of scale-free network. We look at how often the users are active i.e. their activation frequencies. The activation frequencies in the interval of one day are presented in Fig. S1E which is the average over the seven days. The active frequencies are similar to the finding of the works [3, 4]. Most of the users activate once a day. As the users are not regularly active using the App, their disease spreading potential is missed. Our analyzed shows that about 30% users are present one day only in the network.

S1.3 Various network configurations

We have constructed six networks from the above extracted networks. The constructed SPDT network connects 364K individuals and has 6.86M dynamic links. The corresponding SPST network which excludes the indirect transmission links from the SPDT network have connected 264K individuals and has 2.10M dynamic links. We have seen in the Fig. S1F that most of the users stay in the system only 3-4 days on average and then disappear for the remainder of the simulation period. Thus, these networks have low links density and is called Sparse SPDT network (SDT network) and Sparse SPST network (SST network). We reconstruct Dense SPDT network (DDT network) from the SDT network repeating the links from the available days of a user to the missing days for that user. The corresponding Dense SPST network (DST network) is built excluding indirect links from DDT network. Now, the DDT network has 47.11M links connecting 364K users while DST network has 13.24M links connecting 264K users. Thus, the link density increases in the dense network.

The users who are connected with other users through only indirect links in SPDT networks become isolated in SPST networks as indirect links do not exist in SPST networks. Thus, link density reduces in SST network to 7.95 links from 17.86 links of SDT network and 50.15 links for DST network from 129.42 links of DDT network. The underlying social structure is also reshaped since connected users reduce in the SST and DST networks. Thus, we reconstruct LDT and LST networks that maintain the same link densities as that of the DDT networks. In this format, neighbor user's arrival time t'_s of the SPDT links that has only indirect components in DDT networks is set to t_s of host user and a LDT network is obtained. Then, indirect components of links are removed from LDT network to built the LST network which now has the same link density of 129.42 and no isolated user. A summary of the constructed networks is presented in Table 1.

S2 SPDT risk assessment model

S2.1 Mathematical formulation

Our SPDT risk assessment model determines the disease transmission probability for a co-location interactions accounting both direct and indirect transmissions opportunities. Let us consider that an infected individual appearing at a location L and depositing airborne infectious particles (containing a virus) within proximity with a rate g (particles/s). These particles are homogeneously distributed into the air volume V and particle concentration are increased. Simultaneously, particle concentration decays at rate r (proportion/s) from the proximity due to various reduction processes such as particle removal for air exchange from the interaction location, settling down of the particles to the ground and lose of infectivity of particles etc. Thus, the accumulation rate of particles in the proximity can be given by

$$V\frac{\mathrm{d}C}{\mathrm{d}t} = g - rVC \tag{1}$$

where C is the current number of particles in one m^3 of air at L [7]. The particle concentration C_t at time t after the infected individual arrives at L at time t_s is given as

$$\int_0^{C_t} \frac{dC}{g - rVC} = \frac{1}{V}\int_{t_s}^{t} dt$$

This leads to

$$C_t = \frac{g}{rV}\left(1 - e^{-r(t-t_s)}\right) \tag{2}$$

Let us suppose that a susceptible individual arrives at location L at a time t'_s after the infected individual first appeared at L at t_s and left at t_l. If the susceptible individual is concurrently present with the infected individual at L up to time $t'_l < t_l$, the number of particles inhaled by the susceptible individual for this direct link is

$$E_d = \frac{gp}{rV}\int_{t'_s}^{t'_l}\left(1 - e^{-r(t-t_s)}\right) dt$$

where p is the pulmonary rate of the susceptible individual. Thus, we get

$$E_d = \frac{gp}{rV}\left[\left(t'_l + \frac{1}{r}e^{-r(t'_l - t_s)}\right) - \left(t'_s + \frac{1}{r}e^{-r(t'_s - t_s)}\right)\right]$$

$$= \frac{gp}{rV}\left[t'_l - t'_s + \frac{1}{r}e^{-r(t'_l - t_s)} - \frac{1}{r}e^{-r(t'_s - t_s)}\right] \quad (3)$$

If the susceptible individual stays with the infected individual (direct interaction) as well as after the latter leaves L (i.e. $t'_l > t_l$ which creates indirect interaction), it will have both direct and indirect transmission links. The number of particles inhaled by the susceptible individual due to the direct link within the time t'_s and t_l is given by

$$E_d = \frac{gp}{rV}\int_{t'_s}^{t_l}\left(1 - e^{-r(t-t_s)}\right)dt$$

$$= \frac{gp}{rV}\left[t_l - t'_s + \frac{1}{r}e^{-r(t_l - t_s)} - \frac{1}{r}e^{-r(t'_s - t_s)}\right] \quad (4)$$

where t_l is the departing time of infected individual. For the indirect link from time t_l to t'_l, we need to compute the particle concentration during this period which decreases after the infected individual leaves. According to the Equation 2 the particle concentration at time t_l can be given as

$$C_{t_l} = \frac{g}{rV}\left(1 - e^{-r(t_l - t_s)}\right)$$

The particle concentration at time t after the susceptible leaves the proximity at time t_l is given by

$$\frac{dC}{dt} = -Cr$$

Thus, the concentration at time t will be

$$\int_{C_{t_l}}^{C_t}\frac{dC}{C} = -r\int_{t_l}^{t}dt$$

Therefore, the particle concentration C_{t_l} at time t after infected individual leave location at time t_l

$$C_t = C_{t_l}e^{-r(t-t_l)} = \frac{g}{rV}\left(1 - e^{-r(t_l - t_s)}\right)e^{-r(t-t_l)}$$

The susceptible individual inhales particles during the indirect period from t_l to t'_l, quantified by

$$E_i = \int_{t_l}^{t'_l} pC_t dt$$

$$= \frac{gp}{rV}\left(1 - e^{-r(t_l - t_s)}\right)\int_{t_l}^{t'_l} e^{-r(t-t_l)}dt$$

$$E_i = \frac{gp}{Vr^2}\left(1 - e^{-r(t_l - t_s)}\right)\left[1 - e^{-r(t'_l - t_l)}\right] \quad (5)$$

For the situation where susceptible individual is only present for the indirect period at the proximity (i.e. $t'_s > t_l$), the number of inhaled particles for the period from t'_s to t'_l is given

$$= \frac{gp}{rV}\left(1 - e^{-r(t_l - t_s)}\right)\int_{t'_s}^{t'_t} e^{-r(t-t_l)}dt$$

$$= \frac{gp}{Vr^2}\left(1 - e^{-r(t_l - t_s)}\right)\left[e^{-r(t'_s - t_l)} - e^{-r(t'_l - t_l)}\right] \tag{6}$$

To generalize the above intake dose equations into one equation that counts any configuration of SPDT link: direct and/or indirect transmission link, we introduce a link characterizing time t_i parameter. Based on this, we can rewrite the above equations for direct and indirect components as

$$E_d = \frac{gp}{rV}\left[t_i - t'_s + \frac{1}{r}e^{-r(t_i - t_s)} - \frac{1}{r}e^{-r(t'_s - t_s)}\right]$$

$$E_i = \frac{gp}{Vr^2}\left(1 - e^{-r(t_l - t_s)}\right)\left[e^{-r(t_i - t_l)} - e^{-r(t'_l - t_l)}\right]$$

Value of t_i is given as follows: $t_i = t'_l$ for the SPDT links with only direct component, $t_i = t_l$ if SPDT link has both direct and indirect components, and otherwise $t_i = t'_s$. Thus, the total inhaled particles can be given for a susceptible individual who have a SPDT link with an infected individual by

$$E_l = E_d + E_i$$

$$E_l = \frac{gp}{Vr^2}\left[r\left(t_i - t'_s\right) + e^{rt_l}\left(e^{-rt_i} - e^{-rt'_l}\right) + e^{rt_s}\left(e^{-rt'_l} - e^{-rt'_s}\right)\right] \tag{7}$$

The equations determine the received exposure for one SPDT link with an infected individual, comprising both direct and indirect links. In this equation, t_s can not be greater than t'_s as SPDT link is created after infected individual arrive at a location. If $t_s > t'_s$, we have to set $t_s = t'_s$ for calculating appropriate exposure. If a susceptible individual has m SPDT links during an observation period, the total exposure he received is

$$E = \sum_{k=0}^{m} E_l^k$$

where E_l^k is the received exposure for kth link. The probability of infection for intake dose E of infectious particles can be determined by the dose-response relationship as

$$P_I = 1 - e^{-\sigma E} \tag{8}$$

where σ is the infectiousness of the virus to cause infection. This value depends on the disease types and the virus types [8].

S2.2 Characterizing model

The developed risk assessment mode is analyzed here for the influenza like diseases. The influenza diseases are assumed to be transmitted through the airborne routes. Various

practical experiments have been conducted to understand the viral load of influenza A disease in the literature. The experiment of [9] have analyzed breath form the influenza A patients. They found that 65% virus are contained within the $< 5\mu m$ droplets of normal breath and can be airborne and suspended in the air for a long time. During 30 minutes of breathing, patients have generated 140 PFU (plaque-forming unit) virus contained particles. In another experiment of measuring virus in coughs of influenza patients [10], 5-538 PFU viable influenza A virus are found in the samples of six coughs from each patient. If the mid value of range for six coughs is considered as usual, the generated virus for each cough is 269/6 = 44.8 PFU. The mean cough frequency for human is 18 per hour. Therefore, virus generation rate per second by an influenza A infected patient through breathing and coughing will be

$$g = (140/1800) + (44.8 * 18/3600) = 0.304PFU/s$$

Each influenza infected individual can generate 0.34 PFU viable virus per second which are scattered in his close proximity. Experiments show that the intake dose to cause infection in 50% susceptible individuals is in the range 0.69-3.5 PFU for influenza like diseases [11].

The generated virus contained particles are continuously removed from the proximity. The main removal mechanism often comes from the air change rates (ACR) in the proximity. For large scale simulation, it is hard to record air change rate at each interactions. A literature survey is conducted to find the air change rates for various public settings. In the residential areas, air change rates are measured below 1h^{-1}. However, it becomes higher up to 3h^{-1} with opening the windows and doors [12, 13]. In the class room setting, ACR is found from 2 to 6h^{-1} with the median 3h^{-1}. The air exchange rates in the office buildings are 3-4h^{-1}. In the open public places, ACR is varied for a wide range 0.5 to 6h^{-1}. The present particles of proximity also lose their infectivity over time. The other removal mechanism is to settle down on the surfaces. Considering all removal mechanism, we assume, in our study, the upper bound of particles removal rates from interacted proximity can be up to 8h^{-1}. As many studies found the suspended particles remains in the proximity up to several hours. Thus, the lower limit of particles removal rates is considered to be 0.2h^{-1}. Therefore, particles may require 7.5 min to 300 min to be removed from interaction areas after their generation.

Now, the impacts of removal rates for developing virus concentration in the proximity is studied. It is assumed that the virus contained particles disperse up to 20 m in the horizontal direction and 2 m in the vertical direction from the source of infected individual. Thus, the generated infectious particles are scattered in the air volume of $V = 3.14*20*20*2 = 2512$ m^3. In this experiment, we assign $r = \frac{1}{60b}$ to Eqn 7 where b is particle removal time randomly chosen from [7.5-300] min given a median particle removal time r_t. The particle removal rates r is defined with r_t, i.e. particle decay rate r_t means the corresponding particle removal rates r for links drawn from the above process. The particle concentration is analyzed for particle decay rates $r_t = \{10, 20, 30, 40, 50, 60\}$ min while infected individual has stayed up to the 200 minutes ($t_l - t_s = 200$) (see Figure S2a).

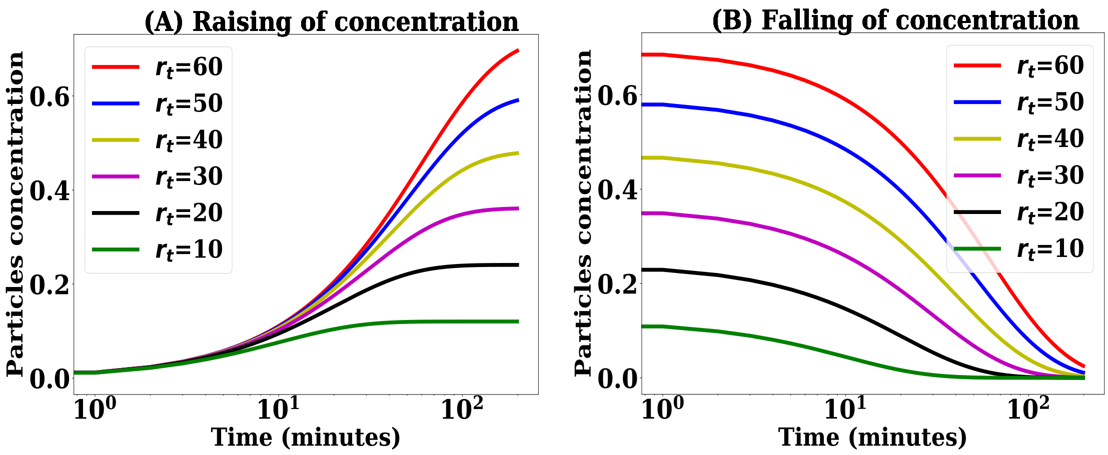

Figure S2: A) increase of the virus concentration when infected individual is present at the proximity and B) decrease of concentration of particles after he leaves the proximity having stayed 200 minutes

The Figure S2b shows the decrease of concentration after infected individual has left the interaction area staying 200 minutes. At the highest removal rate $r_t = 10$ min, the concentration reaches to the steady state very quickly and the concentration of particles is low. Similarly, the concentration drops very quickly after the infected individual leaves the proximity at $r_t = 10$. As the removal rates become lower, the particle concentration increase significantly, but it takes longer time to reach the steady states. They also show slow particle concentrations decay and get long time to remove all particles after infected individual leaves the proximity. Thus, the assumption of arrival of all infected individual at the same time will over estimate the infection risk and this will be severe in the low particle removal rates. On the other hand, the exclusion of exposure during the indirect periods will underestimate the infection risk. For the contacts occurring at locations with lower r, the infection risk will be significantly higher than the contacts occurring at location with higher r.

Calculating infection risk for each contact between infected and susceptible individuals allows to integrate heterogeneities for interaction areas. We estimate the infection risks for 1210 real SPDT contacts between social networking application Momo users over a day from the Beijing city. The contact duration are determined based on the GPS location updates of Momo users. In the estimation of intake doses, we assign $r = \frac{1}{60b}$ to Eqn 7 where b is particle removal time randomly chosen from [7.5-300] min given a median particle removal time r_t. The particle removal rates r is defined with r_t, i.e. particle decay rate r_t means the corresponding particle removal rates r for links drawn from the above process. Susceptible individual has pulmonary rate $p = 1.66 \times 10^{-4} \mathrm{m}^3\mathrm{s}^{-1}$. 1K Momo users are randomly chosen as infected individual and all other users in the network of day one are susceptible individual. Then, if any susceptible individual has contact with the infected individual, their inhaled infectious particles are calculated using Eq.7. Finally, intake dose of infectious particles per minute during a contact is determined as

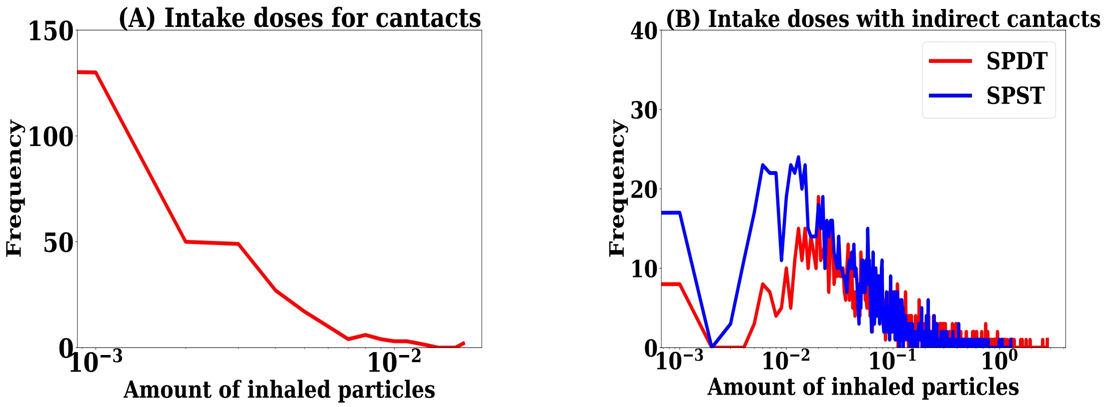

Figure S3: A) Intake dose per minute of contacts between infected and susceptible individuals and B) distribution of intake doses in the proposed model (SPDT) and current models (SPST)

susceptible's stay lengths vary. The distribution of intake dose per minute for 1210 contacts between infected and susceptible individuals are presented in the Figure S3a. The intake doses per minute for contacts have heterogeneous values capturing random distribution of infectious particles in real scenarios. The distribution of received exposures for the contacts between infected and susceptible individuals is also studied. When indirect transmission routes of contacts are accounted, the number of susceptible individuals receiving infectious particles are increasing (see Fig S3b). The amount of intake doses per susceptible individual also increase due to indirect paths (red line). Our proposed model is capable of capturing the lost in estimating the intake dose by the current models.

S3 Simulation and Analysis

We have presented summary of our experiment results in the main text. Here, we explain the results in details.

S3.1 Impacts of particle removal rates

Disease prevalence(I_p): The details of disease prevalence on the both SPST and SPDT model with sparse and dense network connectivity are shown here. The SST network is not capable to grow disease prevalence I_p for any value of r_t. But, the inclusion of indirect links make the SDT network to grow I_p for the decay rates of $r_t \geq 40$ min. For other values of r_t, I_p drops from the initial values but the rate of decline is slower than that of SST network (see Fig. S4). With the high link density, the SPDT model requires very low $r_t \geq 20$ min (Fig. S5). At other values of r_t, I_p starts growing with an initial drop but not leading to strong disease prevalence. On the other hand, DST network has I_p below the initial value. With the weak disease reproduction abilities R, the DST

network could not achieve strong disease prevalence. At $r_t = 10$ min, I_p almost becomes the same in both SPST and SPDT model.

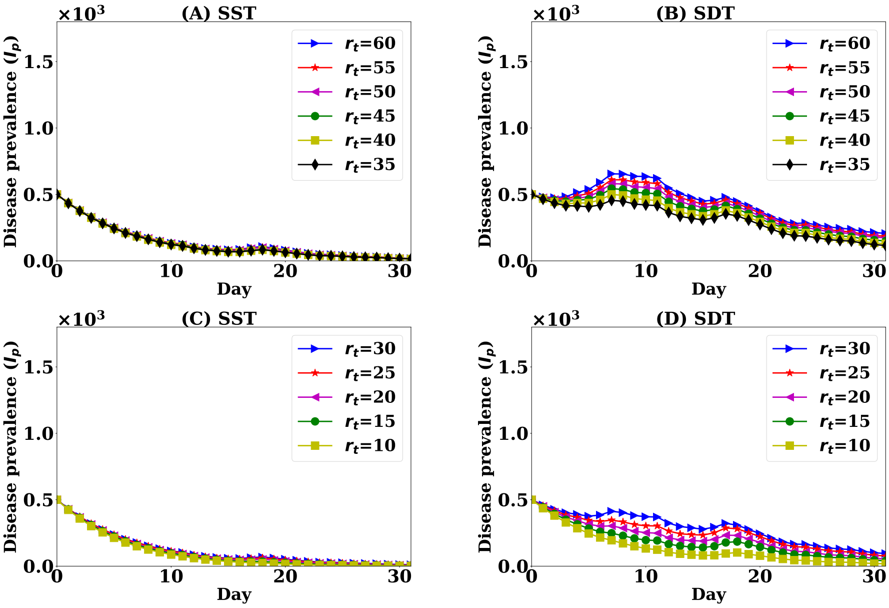

Figure S4: Disease prevalence on the sparse networks: SST and SDT. The upper row for $r_t \geq 35$ min and lower row for $r_t < 35$ min

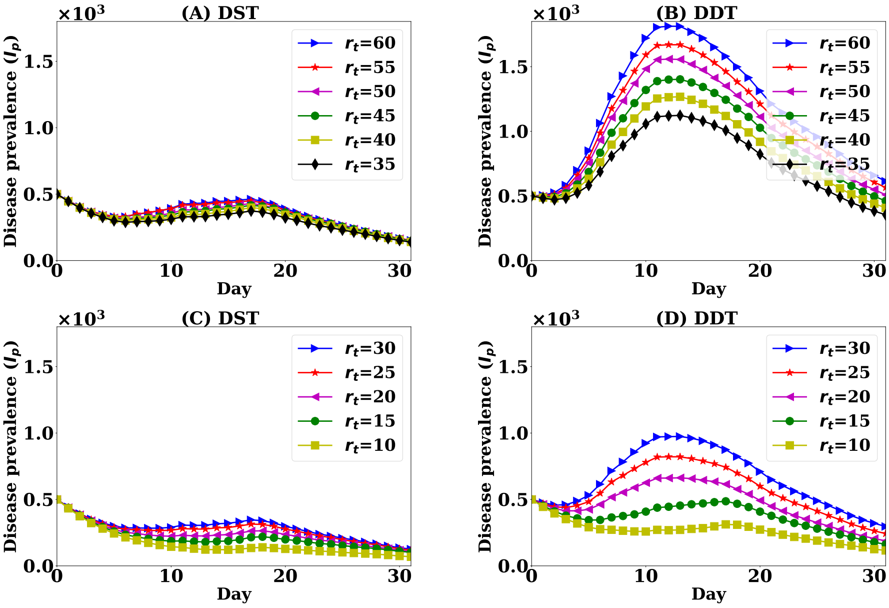

Figure S5: Disease prevalence on the dense networks: DST and DDT. The upper row for $r_t \geq 35$ min and lower row for $r_t < 35$ min

Disease reproduction abilities (R): We have calculated daily disease reproduction abilities R based on the developed equation. As r_t increases, the disease reproduction rate R increases in the both models. But, the increase is more in the SPDT model as the indirect links are strongly affected by r_t. In the sparse networks, there is a sudden increase in R around day 17. This is because the link density around that day increase and infected individuals produce disease strongly during this time. At $r_t = 10$ min, the value of R in SPDT model becomes close to that of SPST model.

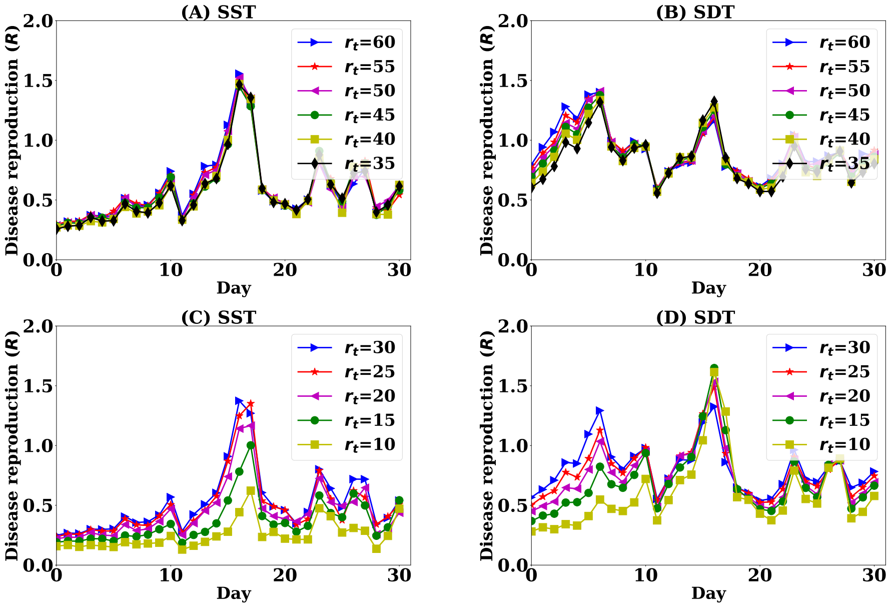

Figure S6: Disease reproduction (R) on the sparse networks: SST and SDT. The upper row for $r_t \geq 35$ min and lower row for $r_t < 35$ min

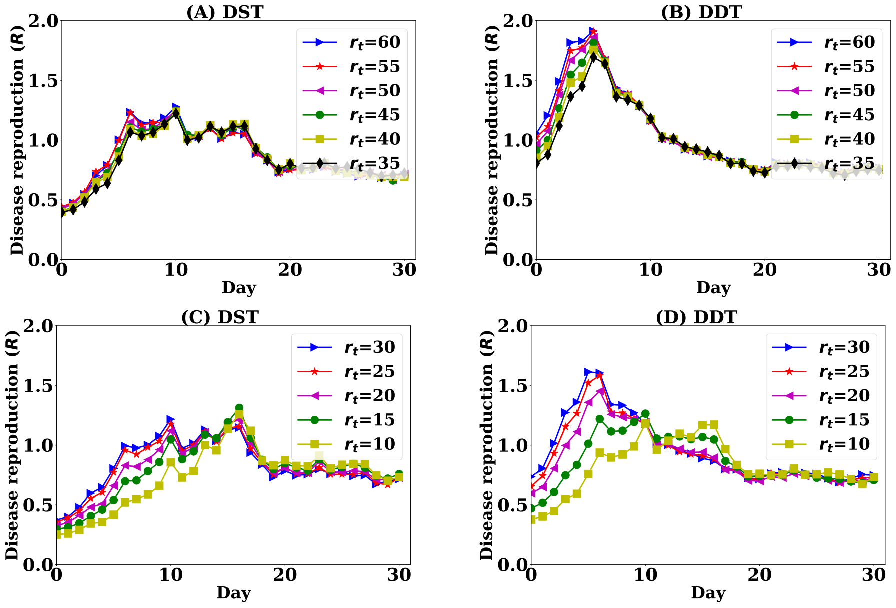

Figure S7: Disease reproduction (R) on the dense networks: DST and DDT. The upper row for $r_t \geq 35$ min and lower row for $r_t < 35$ min

Links per infected individual: We plot the average number of links infected individual sent to the susceptible individuals. It is found that the disease reaches the individuals having a high number of links. As r_t decreases, the average links increases high at later days of simulation in the DST and DDT networks. This is because the disease slowly reaches to the higher degree individual with weak R at low r_t. Thus, higher degree individuals still remain uninfected and get infected later.

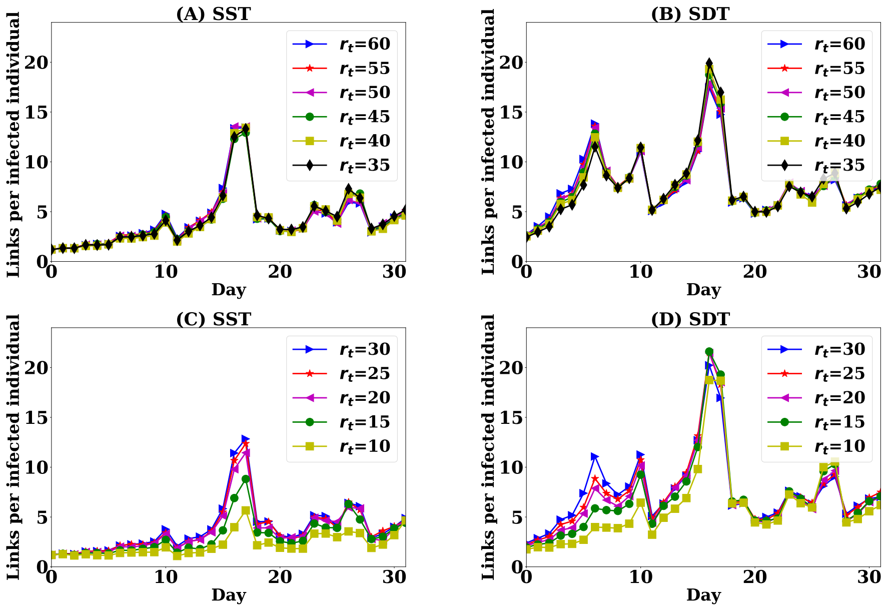

Figure S8: Links per infected individuals on SST and SDT networks. The upper row for $r_t \geq 35$ min and lower row for $r_t < 35$ min

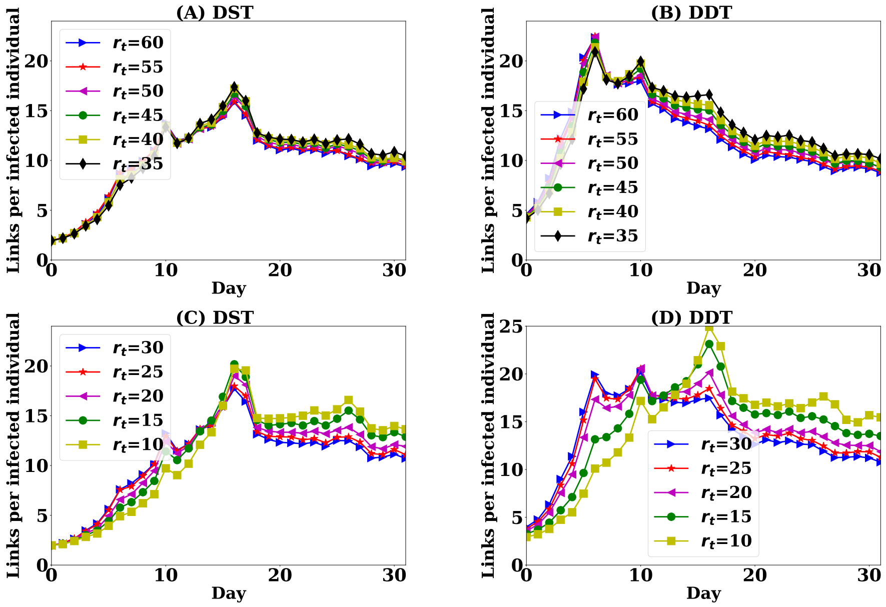

Figure S9: Links per infected individuals on DST and DDT. The upper row for $r_t \geq 35$ min and lower row for $r_t < 35$ min

Infected neighbours per infected individual: Infected individuals also send links to the other infected individuals along with susceptible individuals. This increases as time goes and reduces the ability of causing infection for an infected individual. The DST network and DDT network have the same value although the total infection is highly different. The disease spreads widely in the DDT network comparing to DST network. Thus, infected individuals have less opportunities to interact with other infected individual. In contrast, disease could not spread widely in the DST network due to weak underlying connectivity. Thus, the DST network has a high opportunity to contact other infected individuals. Thus, they have similar rates of infected individuals.

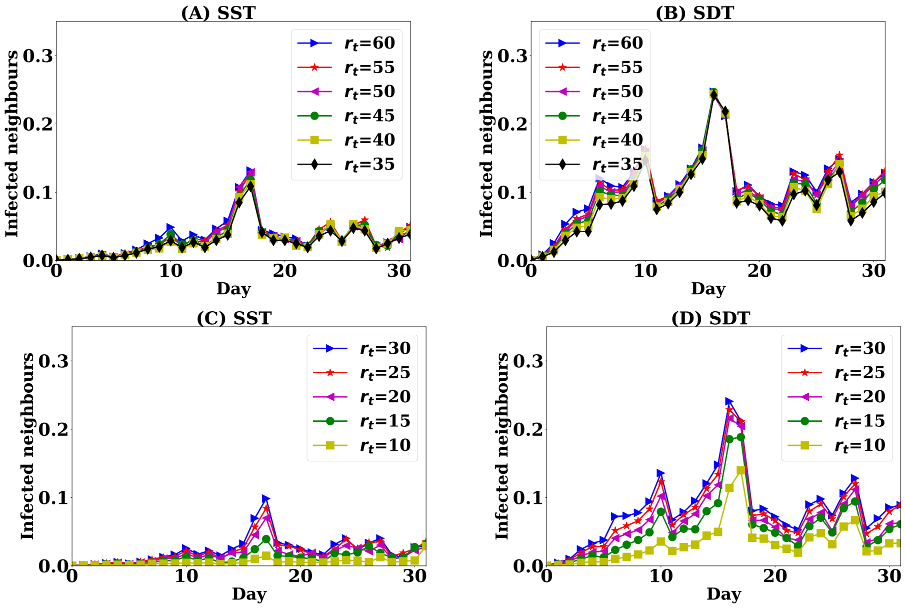

Figure S10: Infected neighbour per infected individual on the sparse networks: SST and SDT. The upper row for $r_t \geq 35$ min and lower row for $r_t < 35$ min

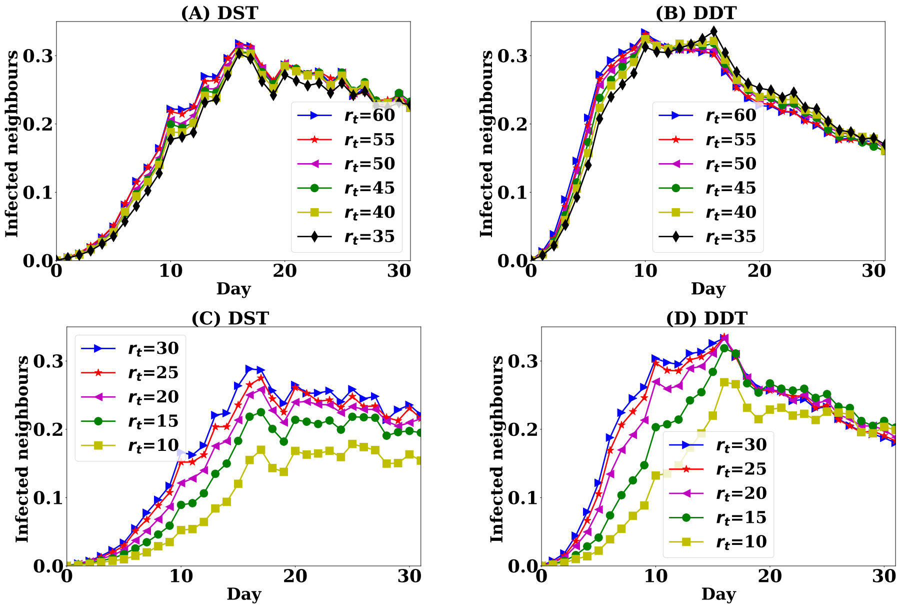

Figure S11: Infected neighbour per infected individual on the dense networks: DST and DDT. The upper row for $r_t \geq 35$ min and lower row for $r_t < 35$ min

S3.2 Impacts of biological disease parameters

Diffusion dynamics with varying infectiousness: Increasing σ reduces the required threshold of r_t. The DDT network is capable to grow I_p for any values of r_t at $\sigma = 0.5$. The DST network has also been capable to grow I_p at this σ for $r_t \geq 40$ min. For the sparse network SDT network, the threshold r_t reduces to 30 min at at $\sigma = 0.5$, but SST could not grow I_p for any value of r_t. Besides, the impacts of σ are more strong in the dense SPDT model.

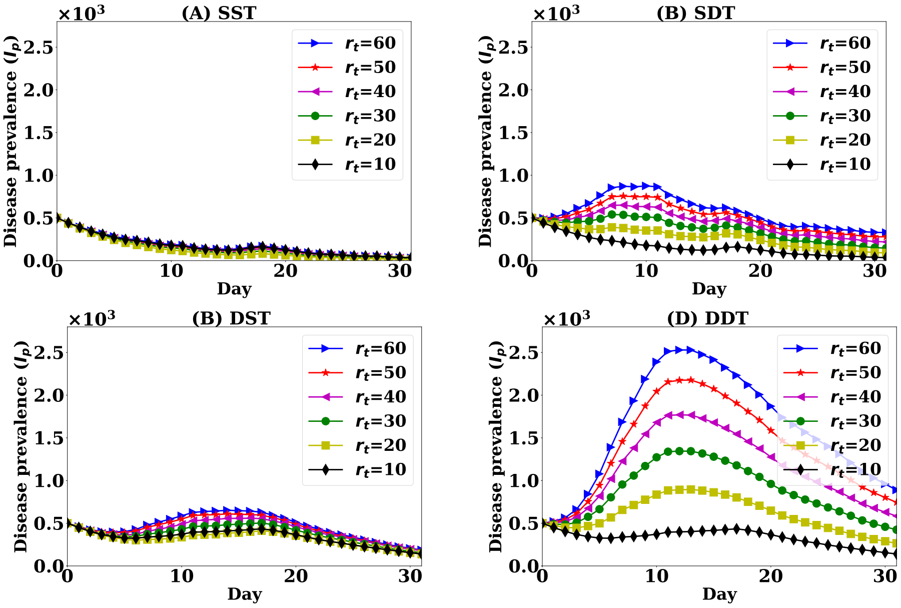

Figure S12: Diffusion dynamics for different networks with $\sigma = 0.4$

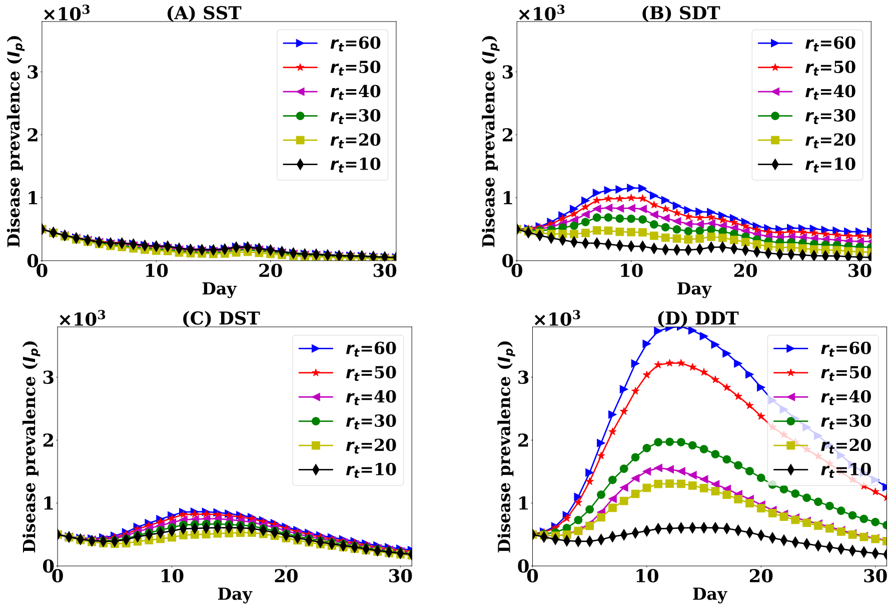

Figure S13: Diffusion dynamics for different networks with $\sigma = 0.5$

Diffusion dynamics with varying infectious periods: We can see that the I_p peak is delayed with the increasing τ. Thus, the total infection is increasing with increasing τ. The impact of τ is strong at the dense network and SPDT model. The interesting behaviour is that the SDT network with $\tau = 5$ days keep the I_p steady over the simulation days. This is because the infected individual are staying for longer time. Thus, the reproduction abilities are increased. As the individuals leave SDT network regularly and new individuals come in, infected individuals have less infected neighbour.

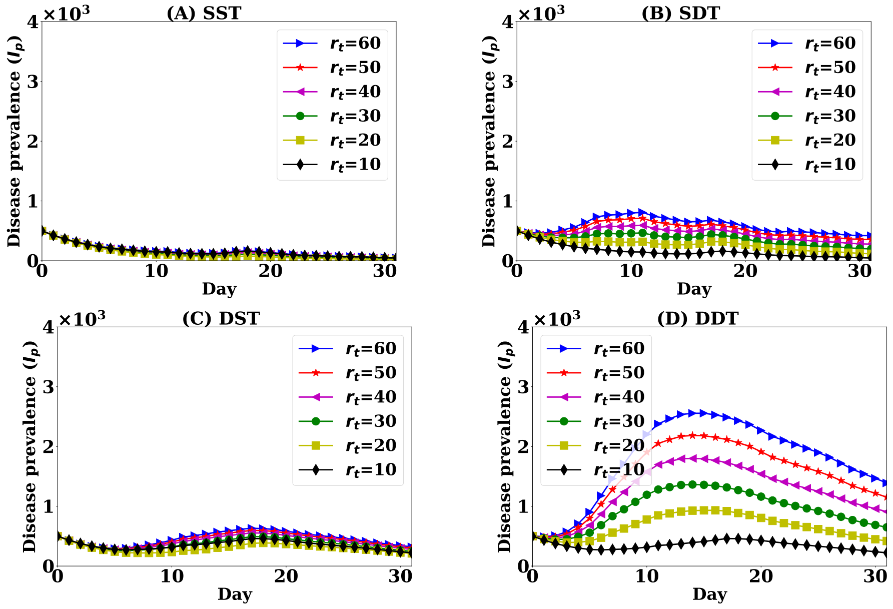

Figure S14: Diffusion dynamics for different networks with $\tau = 4$ day

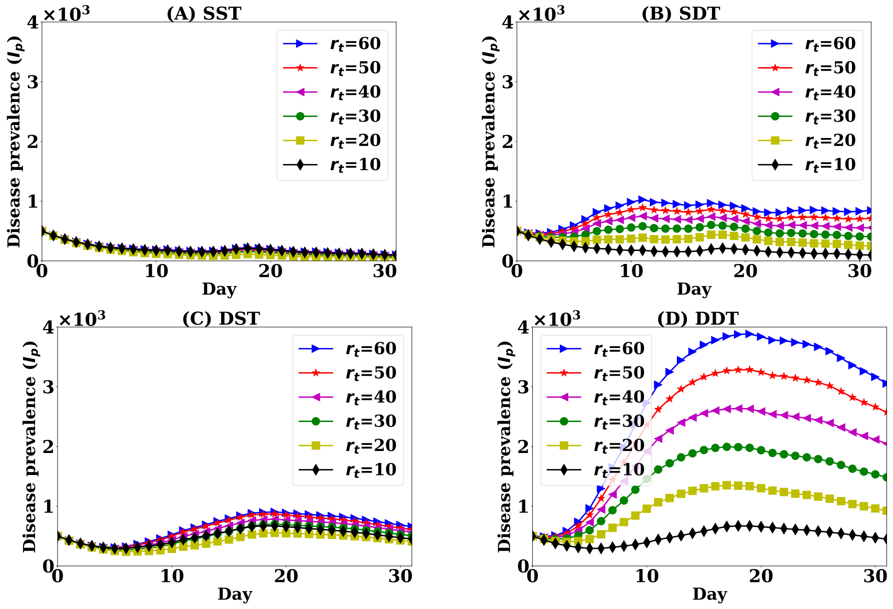

Figure S15: Diffusion dynamics for different networks with $\tau = 5$ day

References

[1] Z. Han, W. Weng, Q. Huang, and S. Zhong, "A risk estimation method for airborne infectious diseases based on aerosol transmission in indoor environment," in *Proceedings of the World Congress on Engineering*, vol. 2, 2014.

[2] M. Shahzamal, R. Jurdak, B. Mans, and F. de Hoog, "Data from: Indirect interactions influence contact network structure and diffusion dynamics," *Github repository*, 2019. [Online]. Available: https://github.com/mszamalbd/Real-SPDT-Contact-Networks

[3] T. M. T. Do and D. Gatica-Perez, "The places of our lives: Visiting patterns and automatic labeling from longitudinal smartphone data," *IEEE Transactions on Mobile Computing*, vol. 13, no. 3, pp. 638–648, 2014.

[4] S. Hasan, C. M. Schneider, S. V. Ukkusuri, and M. C. González, "Spatiotemporal patterns of urban human mobility," *Journal of Statistical Physics*, vol. 151, no. 1-2, pp. 304–318, 2013.

[5] S. H. Strogatz, "Exploring complex networks," *nature*, vol. 410, no. 6825, p. 268, 2001.

[6] L. Muchnik, S. Pei, L. C. Parra, S. D. Reis, J. S. Andrade Jr, S. Havlin, and H. A. Makse, "Origins of power-law degree distribution in the heterogeneity of human activity in social networks," *Scientific reports*, vol. 3, p. 1783, 2013.

[7] C. M. Issarow, N. Mulder, and R. Wood, "Modelling the risk of airborne infectious disease using exhaled air," *Journal of theoretical biology*, vol. 372, pp. 100–106, 2015.

[8] G. Sze To and C. Chao, "Review and comparison between the wells–riley and dose-response approaches to risk assessment of infectious respiratory diseases," *Indoor Air*, vol. 20, no. 1, pp. 2–16, 2010.

[9] J. Yan, M. Grantham, J. Pantelic, P. J. B. de Mesquita, B. Albert, F. Liu, S. Ehrman, D. K. Milton, W. Adamson, B. Beato-Arribas *et al.*, "Infectious virus in exhaled breath of symptomatic seasonal influenza cases from a college community," *Proceedings of the National Academy of Sciences*, p. 201716561, 2018.

[10] W. G. Lindsley, J. D. Noti, F. M. Blachere, R. E. Thewlis, S. B. Martin, S. Othumpangat, B. Noorbakhsh, W. T. Goldsmith, A. Vishnu, J. E. Palmer *et al.*, "Viable influenza a virus in airborne particles from human coughs," *Journal of occupational and environmental hygiene*, vol. 12, no. 2, pp. 107–113, 2015.

[11] R. H. Alford, J. A. Kasel, P. J. Gerone, and V. Knight, "Human influenza resulting from aerosol inhalation," *Proceedings of the Society for Experimental Biology and Medicine*, vol. 122, no. 3, pp. 800–804, 1966.

[12] C. Howard-Reed, L. A. Wallace, and W. R. Ott, "The effect of opening windows on air change rates in two homes," *Journal of the Air & Waste Management Association*, vol. 52, no. 2, pp. 147–159, 2002.

[13] S. Shi, C. Chen, and B. Zhao, "Air infiltration rate distributions of residences in beijing," *Building and Environment*, vol. 92, pp. 528–537, 2015.
